# Supplementary material for: Base editing screens define the genetic landscape of cancer drug resistance mechanisms
Source: Nat Genet. 2024 Oct 18;56(11):2479–92. doi: 10.1038/s41588-024-01948-8 (PMC11549056; doi:10.1038/s41588-024-01948-8)
Supplement: Supplementary file 1 — Supplementary Notes 1–7 and Methods. [file 41588_2024_1948_MOESM1_ESM.pdf]

nature genetics

Supplementary information

https://doi.org/10.1038/s41588-024-01948-8

Base editing screens define the genetic landscape of cancer drug resistance mechanisms

In the format provided by the authors and unedited

Supplementary Information

Supplementary Notes 1-7

Supplementary Methods

Supplementary Note 1

To measure the signal-to-noise of the base editing screens, we summarised the number of hits that were control gRNAs for each screen (Methods). Of the 175 hit-scoring gRNAs from the trametinib screens, 0 were control gRNAs. Of the 81 hit-scoring gRNAs from the dabrafenib plus cetuximab screens, 0 were control gRNAs. Of 40 hit-scoring gRNAs from the pictilisib screens, one was a control gRNA (2.5 %). Of the hit-scoring gRNAs from the sotorasib and adagrasib screens, 3/198 (1.51 %) and 1/224 (0.45 %) were control gRNAs, respectively. Of the hit-scoring gRNAs from the olaparib and niraparib screens, 2/65 (3.08 %) and 0/131 were control gRNAs, respectively. Of the hit-scoring gRNAs from the gefitinib and osimertinib screens, 3/103 (2.91 %) and 0/81 were control gRNAs, respectively.

Supplementary Note 2

For prime editing experiments, we generated PC9 cells deficient in *MLH1* (Extended Data Fig. 5a), which has been shown to increase prime editing efficiency[1,2]. Firstly, we confirmed the installation of EGFR C797S in PC9 *MLH1* KO doxycycline-inducible prime editor cells in the presence of osimertinib (Extended Data Fig. 5b). We then created a focused prime editing gRNA (pegRNA) library of 162 pegRNAs[3] (three pegRNAs per variant) designed to install all amino acid substitutions achievable with SNVs at six residues of interest in EGFR. In arrayed validation experiments, we installed variants in the C-terminal regulatory region of EGFR using a stabilised, engineered prime editing gRNA (epegRNA) design[4] and performed competition assays with WT cells.

Supplementary Note 3

Our sc-SNV-seq approach uses iBAR barcodes[5] in gRNAs to cluster clonal groups of daughter cells with identical genotypes. We generated a focused perturb-seq library which performed as expected in proliferation screens in PC9 cells, displaying a significant correlation in gRNA effect size with larger base editing screens and independently validating drug resistance hits for gefitinib and osimertinib (Extended Data Fig. 7a). Furthermore, HT-29 cells harbouring essential-targeting control gRNAs were depleted from single-cell RNA-seq data relative to non-targeting gRNAs, indicating efficient editing (Extended Data Fig. 7b). In

total, we obtained 27,823 cells with confident gRNA assignments, with an average representation of 63 cells per gRNA for CBE, and 87 cells per gRNA for ABE, excluding essential-targeting controls (Supplementary Tables 5-8).

Supplementary Note 4

In line with perturb-seq data, HT-29 cells with engineered drug resistance variants had significantly reduced B2M and HLA protein expression, with the exception of the MAP2K2 Y134H drug addiction variant (1/5 variants; Extended Data Fig. 9a). We also used base editing to engineer three drug addiction variants into a primary colorectal cancer organoid[6-8], CRC-9. Although drug addiction variants alone did not reduce B2M and HLA expression in this model, we observed a consistent increase in B2M and HLA protein expression following MAPK pathway inhibition with trametinib, in line with previous reports[9-13] (Extended Data Fig. 9b). Induction of B2M and HLA expression with MEK inhibition was significantly attenuated in tumour organoids harbouring drug addiction variants conferring drug resistance (Extended Data Fig. 9b and 9c). Competition assays with GFP^+ WT control organoids showed a proliferation advantage in MAP2K1 S194P and MAP2K2 Y134H harbouring CRC-9 cells, which was enhanced in the presence of MEK inhibition (Extended Data Fig. 9d). We next tested whether modulation of MAPK signalling would directly affect tumour cell killing by patient-derived autologous, anti-tumour T cells. In co-culture experiments, we observed robust and comparable levels of T-cell mediated killing in all tumour organoid lines (Extended Data Fig. 9e). However, pre-treatment with MEK inhibitor significantly enhanced T cell-mediated cancer cell killing relative to controls without T cells, except in tumour organoids harbouring the MAP2K1 S194P drug addiction variant (Extended Data Fig. 9e).

Supplementary Note 5

We generated a progression-free survival (PFS) outcome score – a metric based on the Spearman rank correlation of differentially expressed genes in drug resistant cells from perturb-seq data, and the log-fold change of gene expression changes at 15 days post-treatment versus pre-treatment in patients with PFS >6 months (Supplementary Methods).

Supplementary Note 6

We observed significantly lower editing (43 % median VAF) outside of the activity window, and rare edits (5/137) that were not the expected C>T and A>G transition variants (Extended Data Fig. 10b). These rare variants were consistent with previously reported cytosine deamination by ABE within TCY contexts[14] (where Y denotes pyrimidine).

Supplementary Note 7

To benchmark our resistance variant map, we surveyed COSMIC-curated drug response data[15] and literature relating to the clinical incidence of resistance to drugs analysed in this study[16,17]. This identified 88 amino acid positions in the screened target proteins associated with drug response (Supplementary Table 16, Methods). 85 of these had at least one gRNA predicted to target the amino acid position, 30 of which (35.29 %) had a concordant drug resistance phenotype in our screening dataset specific to the reported drug and gene target. In addition, we observed 252 edits at amino acid positions that had not been previously associated with altered drug response (e.g. PARP1 Y889C, MAP2K1 S194P, BRAF K499E/R; Supplementary Table 17).

Supplementary Methods

Perturb-seq analysis

Processing and quality control

We used Cell Ranger 7.0.1 to obtain UMI counts for gRNA and mRNA and for cell-calling. For quality control, we removed low outliers for the total count, low outliers for the number of detected features and high outliers for the percentage of counts from mitochondrial genes using the scater[18] Bioconductor package (version 1.20.1), obtaining 56,220 cells (ABE data set: 28,718; CBE data set: 27,502) out of 58,673 cells called by Cell Ranger in the first instance.

gRNA calling

We used Cell Ranger to obtain UMI counts for all gRNAs and a robust probabilistic mixture modelling method[19] to distinguish between higher UMI counts that correspond to a gRNA in a cell, versus ambient background counts. We defined two thresholds for UMI counts; a lower threshold – UMI counts below this threshold have probability of more than 90% of being ambient noise; and an upper threshold-UMI counts above this threshold are ambient noise with a probability of less than 10%. gRNAs were called in a cell if their UMI counts were above the upper threshold and no other gRNA had UMI counts between the lower and upper thresholds. Excluding any cells with splice-essential positive control gRNAs, our robust gRNA calling resulted in 27,823 cells with confident gRNA assignment (ABE data set: 15,496, CBE data set: 12,327). The average number of cells per gRNA excluding the splice-essential gRNAs is 86 for ABE and 63 for CBE, where cells with multiple gRNAs are counted towards each of those gRNAs.

iBAR calling and iBAR groups

First, we used Cell Ranger to obtain UMI counts for all possible iBAR barcodes. Then, we assigned iBAR barcodes to all cells with gRNAs, by assigning the iBARs with the n highest UMI counts to a cell with n gRNAs. For downstream analysis, we combined cells with the same gRNA and the same iBAR to iBAR groups, by combining their counts and then normalising for differences in cell sizes and numbers of aggregated cells by means of library normalisation using the scuttle Bioconductor package[18] (version 1.2.1). iBAR groups are therefore small groups of cells with identical genotypes. Averaging across the iBAR groups with more than one cell and treating each iBAR group like a cell in downstream analysis avoids biases in analysis such as false positives or negatives for differential expression (DE) analysis resulting from different numbers of cells with different edits. The use of similar cellular identifiers has been shown to improve accuracy in pooled CRISPR-screens[5,20]. For general scRNA-seq analysis, unmodelled heterogeneity has been identified as a major cause of false positives in DE analysis[21]. For our screen, unlike traditional perturb-seq screens, we were therefore able to account for differences in editing and the clonal structure of the data using the iBARs.

gRNA-level meta-data from the pooled experiments such as variant type (NT controls, canonical drug resistance, driver, drug addiction) were assigned to iBAR groups as follows: if the iBAR group had only one gRNA, then the meta-data of that gRNA was assigned. In the case of several gRNAs, the stronger impact variant type was assigned (driver/drug addiction > canonical drug resistance > control, and iBAR groups with both driver and drug addiction variant gRNAs were discarded from further analysis). We obtained 9,505 iBAR-groups for ABE and 8,171 for CBE. The average number of iBAR groups per gRNA was 56 for ABE and 45 for CBE, including iBAR groups with multiple gRNAs/iBARs. The mean number of cells per iBAR-group was 1.6 for ABE and 1.5 for CBE.

Pathway analysis

We performed pathway analysis using PROGENy for the pathways available with the tool[22]. PROGENy scores are based on a large collection of external publicly available perturbation experiments. We computed a separate PROGENy score for each iBAR group, and mean scores per gRNA. To improve comparability of scores across pathways, we transformed the scores linearly such that their mean and standard deviation on the NT gRNAs was equal to 0 and 1, respectively.

To extend the number of pathways considered, we also used MAYA[23] with the MSigDB Hallmark pathways[24] as input gene lists. For each pathway, MAYA performs PCA on the input data set restricted to the genes of the pathway and selects those pathways and principal components for which there is bimodality in the data. We then used the pathways flagged as bimodal by MAYA in the same way as for the PROGENy-based analysis.

Differential gene expression analysis

We performed differential DE analysis tests at the gRNA level for all gRNAs associated with drug resistance, drug addiction or driver variants with at least 10 iBAR groups uniquely assigned (Supplementary Tables 5-10). The DE tests were performed by comparing iBAR groups containing each gRNA (as unique gRNA in the iBAR group or one of several gRNAs) to the iBAR groups with NT gRNAs only, using the non-parametric Wilcoxon rank sum test, since the iBAR groups cannot be assumed to follow a parametric distribution like a negative Binomial, as they may have been edited and impacted to different degrees by the same gRNA[19]. We also tested whether there was significant DE of PROGENy or MAYA pathway scores between the test gRNAs and NT controls (Extended Data Fig. 8ab and Supplementary Tables 11-14). We identified the gRNAs with impact on the transcriptome as those with at least one gene or pathway with a p-value less than 10^-6, as a measure of a minimum response to the perturbation (13/20 gRNAs for the ABE and 11/17 for the CBE data set). We controlled the expected average false discovery rate for DE testing per gRNA[25]. The number of significantly differentially expressed genes for those gRNAs at expected average FDR of 0.1 ranged from 28 to 2,624.

We also performed DE tests at the level of variant class, comparing drug addiction to canonical drug resistance. Here, we subsampled the same number of iBAR groups for each gRNA (with more than 20 iBAR groups) to obtain a general result unbiased by specific gRNAs

or target genes , using the standard Benjamini-Hochberg correction of false discovery rate across genes and pathway scores[26].

Dimensionality reduction, clustering and cell-cycle analysis

PCA was performed at the iBAR-group level using genes identified as DE at FDR<0.1 for at least one gRNA and with a log2 fold-change of more than 0.25. A UMAP representation was computed based on this PCA. Significance of differences between proportions of variant types was tested for using the standard chi-squared test. Scores for cell-cycle phases were computed using Seurat[27] (version 4.0.6).

Diffusion scores

We computed diffusion scores as described in our previous single-cell CRISPR screen[19], for all gRNAs associated with drug addiction or resistance (ABE) with at least 10 iBAR groups uniquely assigned. The diffusion scores order the iBAR groups in terms of their progression towards the strongest effect driver/drug addiction variants. Diffusion scores were transferred to gRNAs associated with canonical drug resistance from the CBE dataset by mapping each iBAR group from the CBE to the ABE dataset. Similarity between iBAR groups in ABE and CBE was measured in terms of rank correlation restricted to those genes most rank correlated with the diffusion score within the ABE dataset. Then the CBE iBAR group was assigned the mean diffusion score of those five ABE iBAR groups most similar to it.

A number of gRNAs had bimodal diffusion scores, with a cluster of iBAR groups with higher and one with lower diffusion scores. Those gRNAs were identified by applying a mixture of normal distributions with identical variances, using the mclust R package[28] (version 6.1.1). This identified the best number of clusters, with an optimal cluster number of 2 interpreted as bimodality.

Energy distances

Energy distances[29] were computed between targeting and NT gRNAs for all gRNAs with at least 10 iBAR groups uniquely assigned, based on the principal components obtained from all genes differentially expressed for at least one gRNA for the ABE or CBE data set. For comparability between the ABE and CBE data sets, we computed for each data set the median energy distance across NT gRNAs, and divided energy distances for targeting gRNAs by that number for the respective data set.

PFS outcome scores

To compute the PFS outcome scores, we used published differential gene expression results for BRAF-V600E colorectal cancer patients treated with a combination of PD-1, BRAF and MEK inhibition[30]. Tian et al. reported log2-fold gene expression changes at 15 days versus pre-treatment bulked across patients with progression free survival (PFS) > 6 months

and bulked across those with PFS < 6 months. To perform correlation analysis to our data, we identified the genes with an absolute log2-fold expression change of more than 0.25 and adjusted p-value of less than 0.1 for either the data for PFS > 6 months or PFS < 6 months (2,148 genes). Then, based on this subset of genes, we used Spearman rank correlation to correlate the log2-fold expression changes for PFS > 6 months reported in Tian et al with the log2-fold changes from our DE testing results for the gRNAs (PFS outcome scores).

Supplementary References

1. Chen, P.J. *et al.* Enhanced prime editing systems by manipulating cellular determinants of editing outcomes. *Cell* **184**, 5635-5652 e29 (2021).
2. Ferreira da Silva, J. *et al.* Prime editing efficiency and fidelity are enhanced in the absence of mismatch repair. *Nat Commun* **13**, 760 (2022).
3. Mathis, N. *et al.* Predicting prime editing efficiency and product purity by deep learning. *Nat Biotechnol* (2023).
4. Nelson, J.W. *et al.* Engineered pegRNAs improve prime editing efficiency. *Nat Biotechnol* **40**, 402-410 (2022).
5. Zhu, S. *et al.* Guide RNAs with embedded barcodes boost CRISPR-pooled screens. *Genome Biol* **20**, 20 (2019).
6. Cattaneo, C.M. *et al.* Tumor organoid-T-cell coculture systems. *Nat Protoc* **15**, 15-39 (2020).
7. Coelho, M.A. *et al.* Base editing screens map mutations affecting interferon-gamma signaling in cancer. *Cancer Cell* (2023).
8. Dijkstra, K.K. *et al.* Generation of Tumor-Reactive T Cells by Co-culture of Peripheral Blood Lymphocytes and Tumor Organoids. *Cell* **174**, 1586-1598 e12 (2018).
9. El-Jawhari, J.J. *et al.* Blocking oncogenic RAS enhances tumour cell surface MHC class I expression but does not alter susceptibility to cytotoxic lymphocytes. *Mol Immunol* **58**, 160-8 (2014).
10. Ebert, P.J.R. *et al.* MAP Kinase Inhibition Promotes T Cell and Anti-tumor Activity in Combination with PD-L1 Checkpoint Blockade. *Immunity* **44**, 609-621 (2016).
11. Frederick, D.T. *et al.* BRAF inhibition is associated with enhanced melanoma antigen expression and a more favorable tumor microenvironment in patients with metastatic melanoma. *Clin Cancer Res* **19**, 1225-31 (2013).
12. Mimura, K. *et al.* The MAPK pathway is a predominant regulator of HLA-A expression in esophageal and gastric cancer. *J Immunol* **191**, 6261-72 (2013).
13. Sers, C. *et al.* Down-regulation of HLA Class I and NKG2D ligands through a concerted action of MAPK and DNA methyltransferases in colorectal cancer cells. *Int J Cancer* **125**, 1626-39 (2009).
14. Arbab, M. *et al.* Determinants of Base Editing Outcomes from Target Library Analysis and Machine Learning. *Cell* **182**, 463-480 e30 (2020).
15. Tate, J.G. *et al.* COSMIC: the Catalogue Of Somatic Mutations In Cancer. *Nucleic Acids Res* **47**, D941-D947 (2019).
16. Awad, M.M. *et al.* Acquired Resistance to KRAS(G12C) Inhibition in Cancer. *N Engl J Med* **384**, 2382-2393 (2021).
17. Brammeld, J.S. *et al.* Genome-wide chemical mutagenesis screens allow unbiased saturation of the cancer genome and identification of drug resistance mutations. *Genome Res* **27**, 613-625 (2017).
18. McCarthy, D.J., Campbell, K.R., Lun, A.T. & Wills, Q.F. Scater: pre-processing, quality control, normalization and visualization of single-cell RNA-seq data in R. *Bioinformatics* **33**, 1179-1186 (2017).

19. Cooper, S.E. *et al.* scSNV-seq: high-throughput phenotyping of single nucleotide variants by coupled single-cell genotyping and transcriptomics. *Genome Biol* **25**, 20 (2024).
20. Michlits, G. *et al.* CRISPR-UMI: single-cell lineage tracing of pooled CRISPR-Cas9 screens. *Nat Methods* **14**, 1191-1197 (2017).
21. Squair, J.W. *et al.* Confronting false discoveries in single-cell differential expression. *Nat Commun* **12**, 5692 (2021).
22. Schubert, M. *et al.* Perturbation-response genes reveal signaling footprints in cancer gene expression. *Nat Commun* **9**, 20 (2018).
23. Landais, Y. & Vallot, C. Multi-modal quantification of pathway activity with MAYA. *Nat Commun* **14**, 1668 (2023).
24. Liberzon, A. *et al.* The Molecular Signatures Database (MSigDB) hallmark gene set collection. *Cell Syst* **1**, 417-425 (2015).
25. Benjamini, Y. & Bogomolov, M. Selective Inference on Multiple Families of Hypotheses. *Journal of the Royal Statistical Society Series B: Statistical Methodology* **76**, 297-318 (2014).
26. Benjamini, Y. & Hochberg, Y. Controlling the False Discovery Rate: A Practical and Powerful Approach to Multiple Testing. *Journal of the Royal Statistical Society: Series B (Methodological)* **57**, 289-300 (1995).
27. Butler, A., Hoffman, P., Smibert, P., Papalexi, E. & Satija, R. Integrating single-cell transcriptomic data across different conditions, technologies, and species. *Nat Biotechnol* **36**, 411-420 (2018).
28. Scrucca, L., Fop, M., Murphy, T.B. & Raftery, A.E. mclust 5: Clustering, Classification and Density Estimation Using Gaussian Finite Mixture Models. *R J* **8**, 289-317 (2016).
29. Replogle, J.M. *et al.* Mapping information-rich genotype-phenotype landscapes with genome-scale Perturb-seq. *Cell* **185**, 2559-2575 e28 (2022).
30. Tian, J. *et al.* Combined PD-1, BRAF and MEK inhibition in BRAF(V600E) colorectal cancer: a phase 2 trial. *Nat Med* **29**, 458-466 (2023).
