# Supplementary material for: Base editing screens define the genetic landscape of cancer drug resistance mechanisms
Source: Nat Genet. 2024 Oct 18;56(11):2479–92. doi: 10.1038/s41588-024-01948-8 (PMC11549056; doi:10.1038/s41588-024-01948-8)
Supplement: Supplementary file 2 — Reporting Summary [file 41588_2024_1948_MOESM2_ESM.pdf]

nature portfolio

Corresponding author(s): Mathew Garnett and Matthew Coelho

Last updated by author(s): Aug 19, 2024

Reporting Summary

Nature Portfolio wishes to improve the reproducibility of the work that we publish. This form provides structure for consistency and transparency in reporting. For further information on Nature Portfolio policies, see our Editorial Policies and the Editorial Policy Checklist.

Statistics

For all statistical analyses, confirm that the following items are present in the figure legend, table legend, main text, or Methods section.

n/a	Confirmed	
☐	☒	The exact sample size (*n*) for each experimental group/condition, given as a discrete number and unit of measurement
☐	☒	A statement on whether measurements were taken from distinct samples or whether the same sample was measured repeatedly
☐	☒	The statistical test(s) used AND whether they are one- or two-sided<br>*Only common tests should be described solely by name; describe more complex techniques in the Methods section.*
☐	☒	A description of all covariates tested
☐	☒	A description of any assumptions or corrections, such as tests of normality and adjustment for multiple comparisons
☐	☒	A full description of the statistical parameters including central tendency (e.g. means) or other basic estimates (e.g. regression coefficient) AND variation (e.g. standard deviation) or associated estimates of uncertainty (e.g. confidence intervals)
☐	☒	For null hypothesis testing, the test statistic (e.g. F, t, r) with confidence intervals, effect sizes, degrees of freedom and P value noted<br>*Give P values as exact values whenever suitable.*
☒	☐	For Bayesian analysis, information on the choice of priors and Markov chain Monte Carlo settings
☐	☒	For hierarchical and complex designs, identification of the appropriate level for tests and full reporting of outcomes
☐	☒	Estimates of effect sizes (e.g. Cohen's *d*, Pearson's *r*), indicating how they were calculated

Our web collection on statistics for biologists contains articles on many of the points above.

Software and code

Policy information about availability of computer code

Data collection	For flow cytometry, we used FACSDiva software (version 9; BD Biosciences).
Data analysis	Code used to analyse base editing screens can be found on GitHub here: https://github.com/MatthewACoelho/Res1_analysis. Code used to analyse the single-cell screens can be found here: https://github.com/MarioniLab/BE_perturb_seq_drug_resistance.<br>In amplicon sequencing analysis we used BCFtools (version 1.20) and vaf correct (version 5.4.0).<br>In perturb-seq analysis we used scater (version 1.20.1) and Seurat (version 4.0.6) , scuttle (version 1.2.1) and mclust R (version 6.1.1).<br>For visualization of crystal structures, we used PyMOL (version 2.4.1), for graphs we used GraphPad Prism (version 8) or R ggplot2 (version 3.3.0).<br>For flow cytometry analysis we used FlowJo (version 10) or FCS Express (version 7).

For manuscripts utilizing custom algorithms or software that are central to the research but not yet described in published literature, software must be made available to editors and reviewers. We strongly encourage code deposition in a community repository (e.g. GitHub). See the Nature Portfolio guidelines for submitting code & software for further information.

Data

Policy information about availability of data

All manuscripts must include a data availability statement. This statement should provide the following information, where applicable:

- Accession codes, unique identifiers, or web links for publicly available datasets
- A description of any restrictions on data availability
- For clinical datasets or third party data, please ensure that the statement adheres to our policy

SSequencing data are deposited on ENA and EGA and accessions are described in Supplementary Table 8 (ERP146490, ERP148732, ERP144241, ERP141719, ERP156437, EGAS00001006683, EGAS00001006170, EGAS00001006169, EGAS00001006093, EGAS00001006092, EGAS00001006091) and can be found here: https://www.ebi.ac.uk/ena/browser/home and here, https://ega-archive.org/studies/. All genomic indexing is relative to GRCh38 genome assembly https://www.ensembl.org/Homo_sapiens/Info/Index. COSMIC variants were downloaded in February 2024 version 99 https://cancer.sanger.ac.uk/cosmic/download/cosmic. Unprocessed Western blots are available as Source Data and screening data are available in Supplementary Table 2. Screen z-scores are available on the MAVE database103 (urn:mavedb:00001204).

Research involving human participants, their data, or biological material

Policy information about studies with human participants or human data. See also policy information about sex, gender (identity/presentation), and sexual orientation and race, ethnicity and racism.

Reporting on sex and gender	N/A
Reporting on race, ethnicity, or other socially relevant groupings	N/A
Population characteristics	N/A
Recruitment	N/A
Ethics oversight	N/A

Note that full information on the approval of the study protocol must also be provided in the manuscript.

Field-specific reporting

Please select the one below that is the best fit for your research. If you are not sure, read the appropriate sections before making your selection.

☒ Life sciences ☐ Behavioural & social sciences ☐ Ecological, evolutionary & environmental sciences

For a reference copy of the document with all sections, see nature.com/documents/nr-reporting-summary-flat.pdf

Life sciences study design

All studies must disclose on these points even when the disclosure is negative.

Sample size	No statistical methods were used for sample size determination. Base editing screens gave significant effect sizes for gRNAs that were independently validated as functional. We analysed the effects of all possible variants that could be installed with CBE and ABE in the 11 cancer genes studied, which constituted a library of 22,816 gRNAs.
Data exclusions	We excluded gRNAs that had more than two perfect matches in the GRCh38 human genome (n=134), and gRNAs with < 100 read counts in the plasmid or any time 0 sample in the screens (n=27). For the few hit gRNAs with two perfect matches in the genome, we confirmed there was only one exonic target. We also excluded gRNAs specifically in the MHH-ES-1 screen that had a 10-fold read count difference between replicates (n=118) from downstream analysis of the MHH-ES-1 screens.
Replication	All experiments were performed independently on a separate day at least twice, including CRISPR and base editing screens, as stated in the figure legends. All attempts to repeat experiments were successful.
Randomization	No randomisation was performed. Base editing screens are unbiased, pooled experiments where sample preparation is identical. For other experiments, randomisation was not performed as all cell culture experiments were performed under identical conditions apart from the experimental perturbation.
Blinding	No blinding was performed. Base editing screens are unbiased, pooled experiments where sample preparation is identical. The investigators where unbiased towards the screening data analysis, as this was a hypothesis-generating experiment without prior assumptions of variant effect. For other experiments, blinding was not performed as all cell culture experiments were performed under identical conditions apart from the experimental perturbation.

Reporting for specific materials, systems and methods

We require information from authors about some types of materials, experimental systems and methods used in many studies. Here, indicate whether each material, system or method listed is relevant to your study. If you are not sure if a list item applies to your research, read the appropriate section before selecting a response.

Materials & experimental systems

n/a	Involved in the study
☐	☒ Antibodies
☐	☒ Eukaryotic cell lines
☒	☐ Palaeontology and archaeology
☒	☐ Animals and other organisms
☒	☐ Clinical data
☒	☐ Dual use research of concern
☒	☐ Plants

Methods

n/a	Involved in the study
☒	☐ ChIP-seq
☐	☒ Flow cytometry
☒	☐ MRI-based neuroimaging

Antibodies

Antibodies used

Western blotting primary antibodies: EGFR total (1068 epitope, #2232, 1:1,000 dilution), p-EGFR (1148 region, #4404, 1:1,000 dilution), b-actin (#4970, 1:1,000 dilution), p-ERK (#9101, 1:1,000 dilution), ERK total (#9102, 1:1,000 dilution) (Cell Signaling Technology), EGFR epitope 1020-1046 (#610017 BD Biosciences, 1:1,000 dilution), PARP1 (#9532, clone 46D11, 1:1,000 dilution), lamin A/C (#2032, 1:1,000 dilution), histone H3 (#3638, clone 96C10, 1:1,000 dilution) (all from Cell Signaling Technology).

Secondary antibodies (anti-mouse and ant-rabbit) were conjugated to horseradish peroxidase (#31460 and #31430, Thermo Fisher Scientific, 1:5,000 dilution).

Flow cytometry: anti-EGFR-FITC antibody (#MA5-28104, clone ICR10, Thermo Fisher Scientific, 1:100 dilution).

Validation

No additional validation was performed for these commercially available antibodies but they have been validated by the vendors. Datasheets including validation, citations and application notes can be found here:
https://www.cellsignal.com
https://www.thermofisher.com/uk/en/home/life-science/antibodies/primary-antibodies.html?icid=ab-search-primary-icons

Eukaryotic cell lines

Policy information about cell lines and Sex and Gender in Research

Cell line source(s)	H23 (NCI), PC9 (RIKEN), HT-29 (NCI), MHH-ES-1 (DSMZ), HEK293T (ATCC)
Authentication	All cell models used in this study (H23, PC9, HT-29, MHH-ES-1, HEK293T) were STR profiled in accordance with authentication guidelines.
Mycoplasma contamination	All cell models used in this study were routinely verified as mycoplasma-free.
Commonly misidentified lines (See ICLAC register)	None used in this study.

Plants

Seed stocks

Report on the source of all seed stocks or other plant material used. If applicable, state the seed stock centre and catalogue number. If plant specimens were collected from the field, describe the collection location, date and sampling procedures.

Novel plant genotypes

Describe the methods by which all novel plant genotypes were produced. This includes those generated by transgenic approaches, gene editing, chemical/radiation-based mutagenesis and hybridization. For transgenic lines, describe the transformation method, the number of independent lines analyzed and the generation upon which experiments were performed. For gene-edited lines, describe the editor used, the endogenous sequence targeted for editing, the targeting guide RNA sequence (if applicable) and how the editor was applied.

Authentication

Describe any authentication procedures for each seed stock used or novel genotype generated. Describe any experiments used to assess the effect of a mutation and, where applicable, how potential secondary effects (e.g. second site T-DNA insertions, mosiacism, off-target gene editing) were examined.

Flow Cytometry

Plots

Confirm that:

☒ The axis labels state the marker and fluorochrome used (e.g. CD4-FITC).

☒ The axis scales are clearly visible. Include numbers along axes only for bottom left plot of group (a 'group' is an analysis of identical markers).

☒ All plots are contour plots with outliers or pseudocolor plots.

☒ A numerical value for number of cells or percentage (with statistics) is provided.

Methodology

Sample preparation	PC9 cells were harvested by trypsinisation and washed in FACS buffer (0.5 % FCS< 2 mM EDTA in PBS) before staining with anti-EGFR-FITC antibody (#MA5-28104, Thermo Fisher Scientific) for 25 min on ice in the dark. Cells were washed twice in FACS buffer, incubated with DAPI (1 µg/ml, Thermo Fisher Scientific) before filtering through a nylon mesh cell strainer, and analysis on an LSRFortsessa (BD Biosciences).
Instrument	LSRFortessa; BD Biosciences
Software	Flow cytometry data were acquired using FACSDiva software (version 9, BD Biosciences), and analysed using FlowJo (version 10) or FCS Express (version 7).
Cell population abundance	No verification of post-sort abundance was performed.
Gating strategy	Gating of PC9 cells was based on FSC-A vs SSC-A (cells), FSC-A vs SSC-W (singlets), DAPI vs FSC-A (viable), mApple vs BFP (base editor construct and gRNA construct). A histogram of EGFR-FITC expression in this cell population is shown in Figure 6d. Similarly, Supplementary 9c shows the gating strategy for CRC9 cells stained for MHC-I and B2M.

☒ Tick this box to confirm that a figure exemplifying the gating strategy is provided in the Supplementary Information.
