# Supplementary figures and images for: Base editing screens define the genetic landscape of cancer drug resistance mechanisms
Source: Nat Genet. 2024 Oct 18;56(11):2479–92. doi: 10.1038/s41588-024-01948-8 (PMC11549056; doi:10.1038/s41588-024-01948-8)

Figure 2e

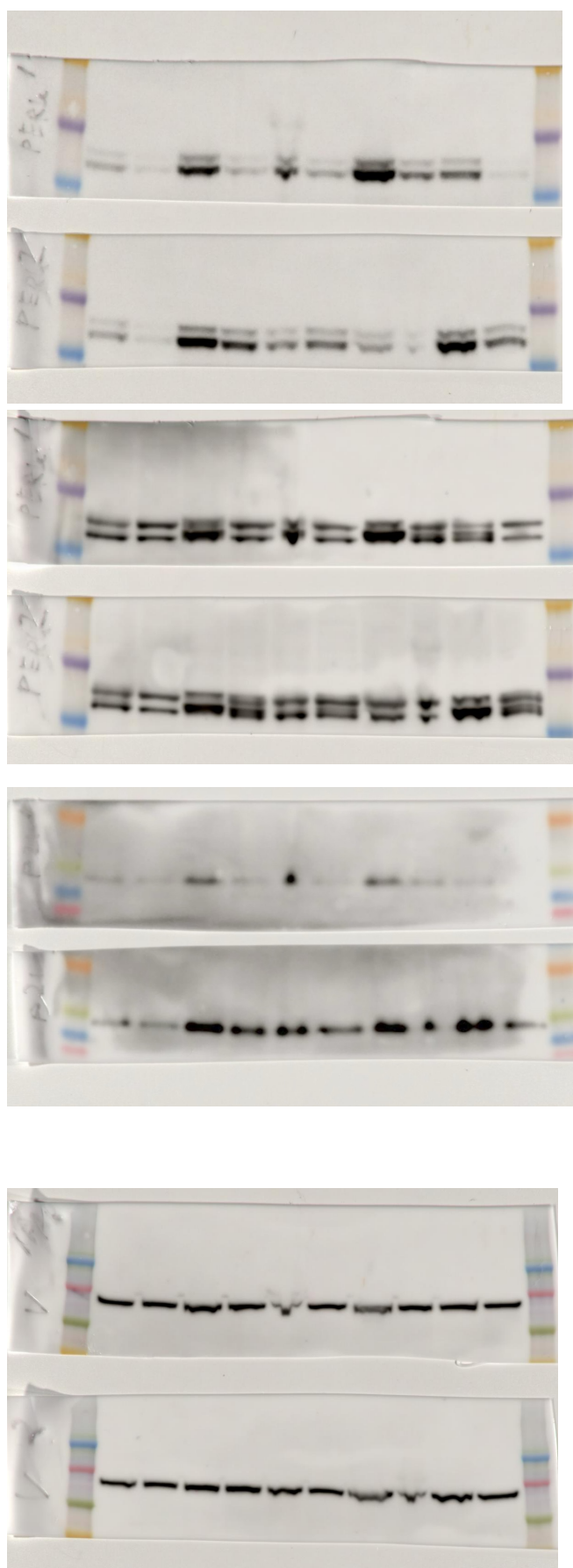

Supplement: Supplementary file 5 — Unprocessed blots. [file 41588_2024_1948_MOESM5_ESM.pdf]

Figure 6c

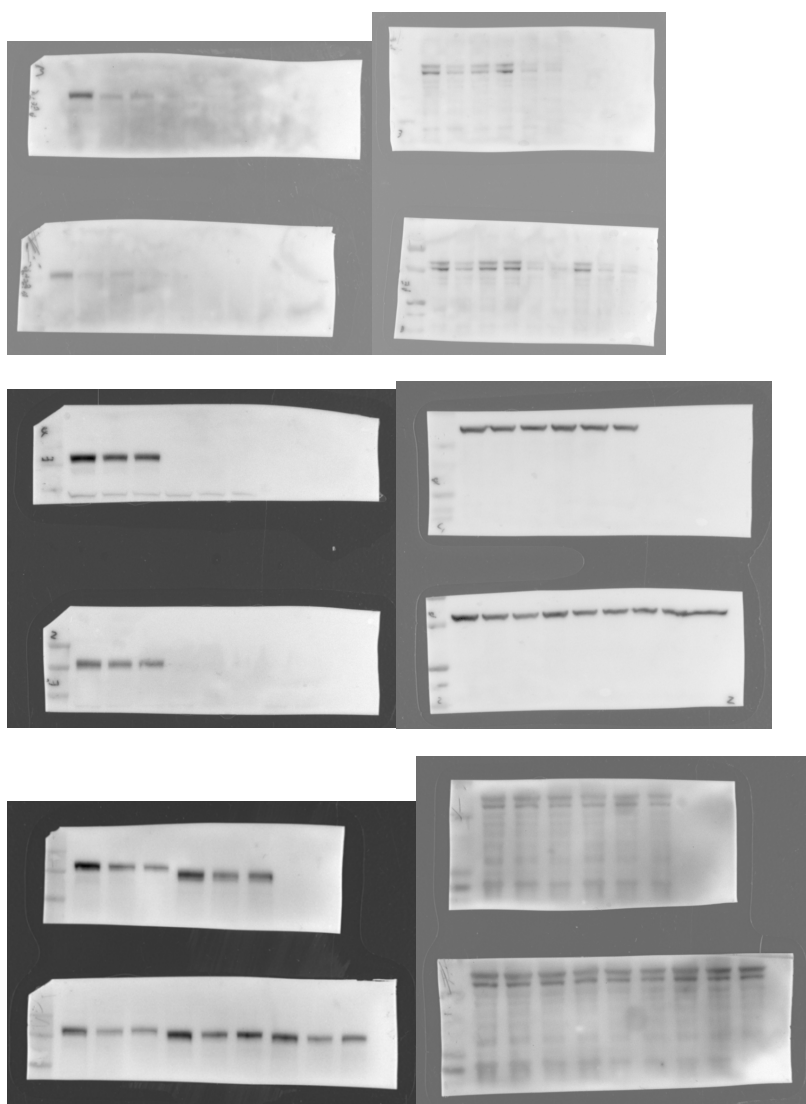

Supplement: Supplementary file 6 — Unprocessed blots. [file 41588_2024_1948_MOESM6_ESM.pdf]

Extended Data Figure 3a

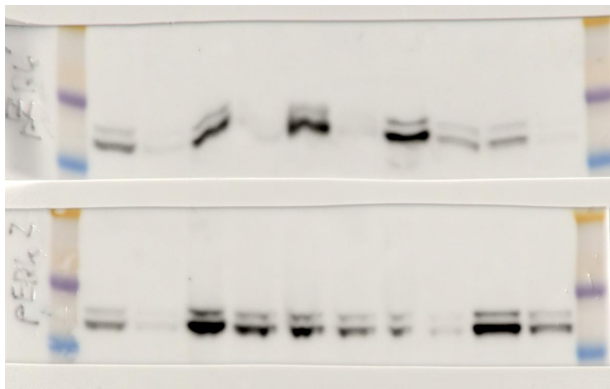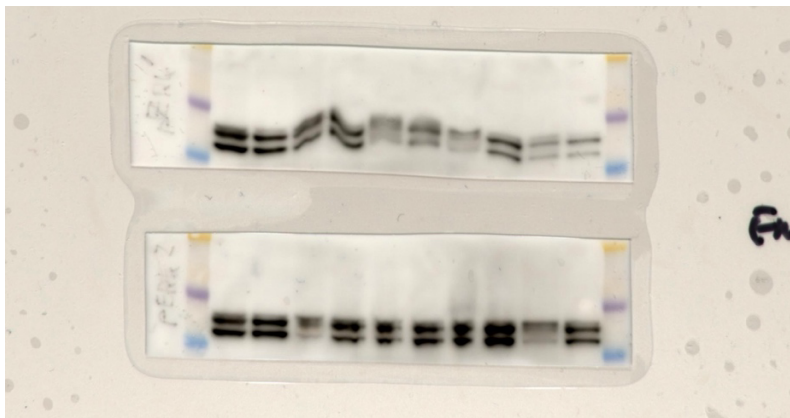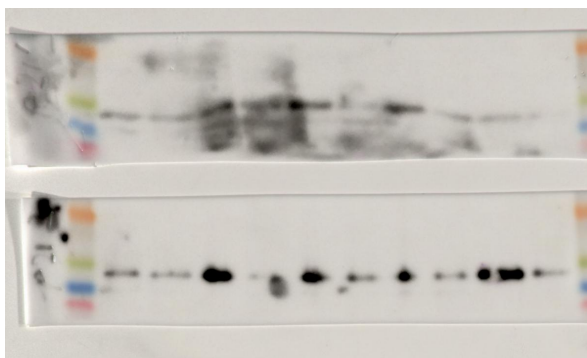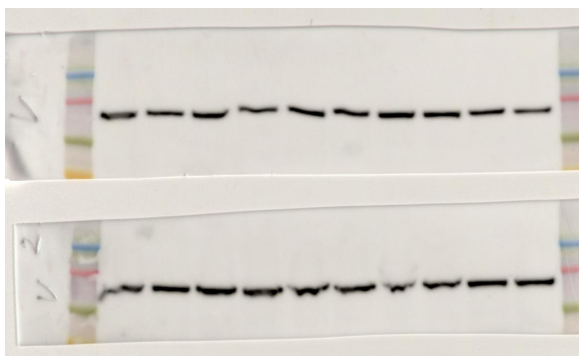

Supplement: Supplementary file 7 — Unprocessed blots. [file 41588_2024_1948_MOESM7_ESM.pdf]

Extended Data Figure 4d

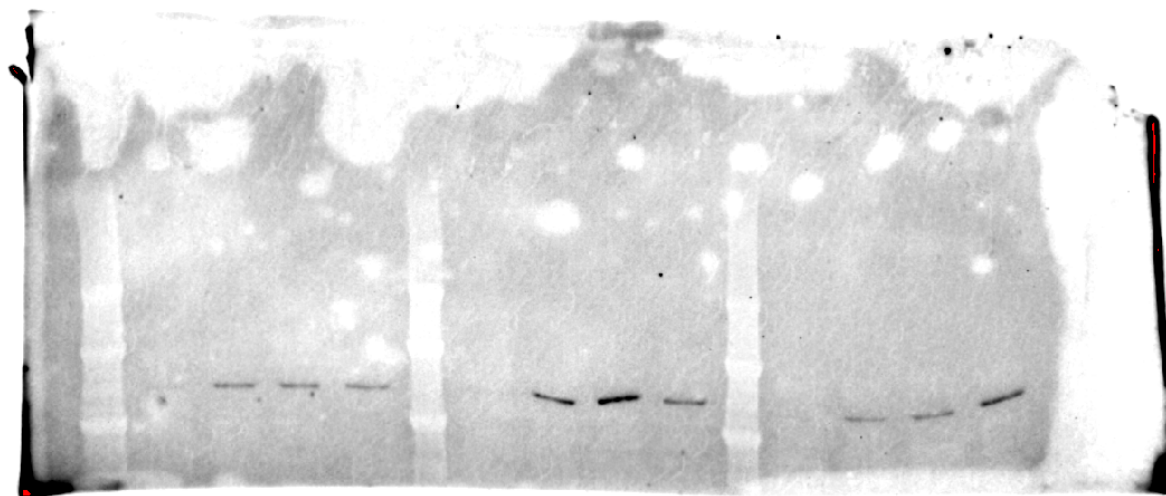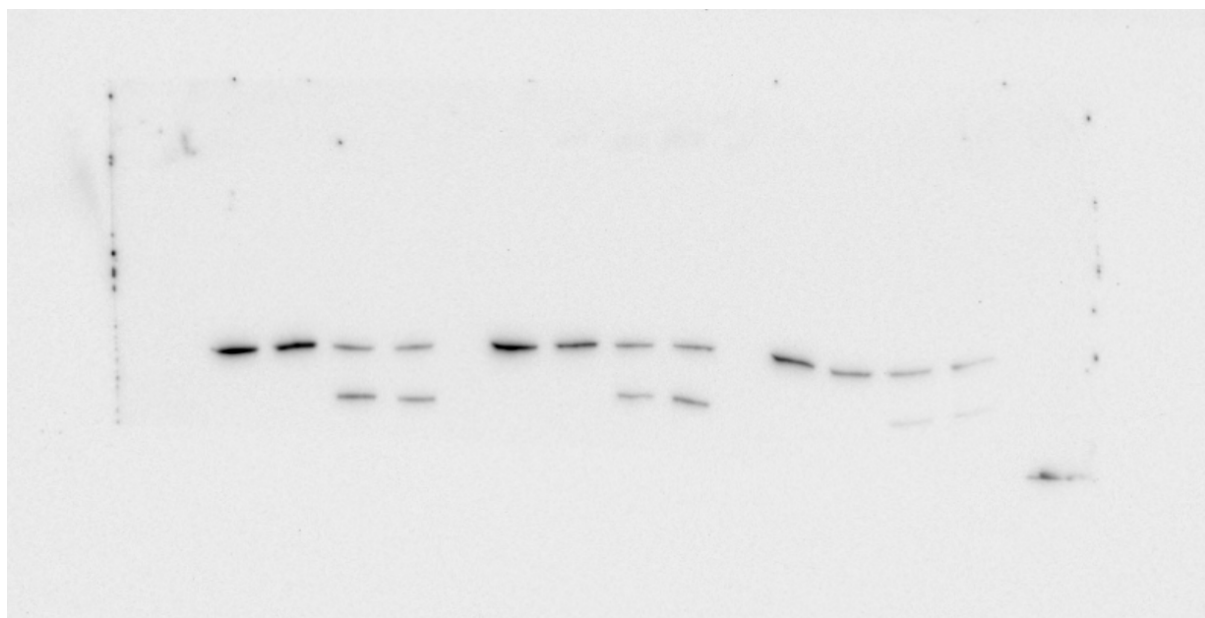

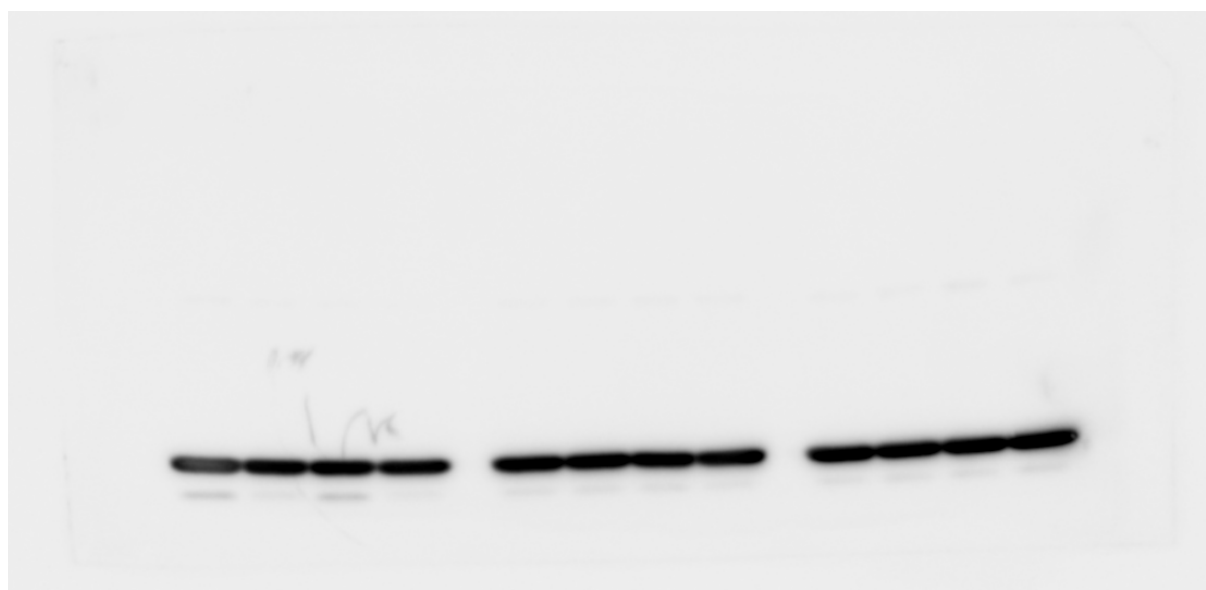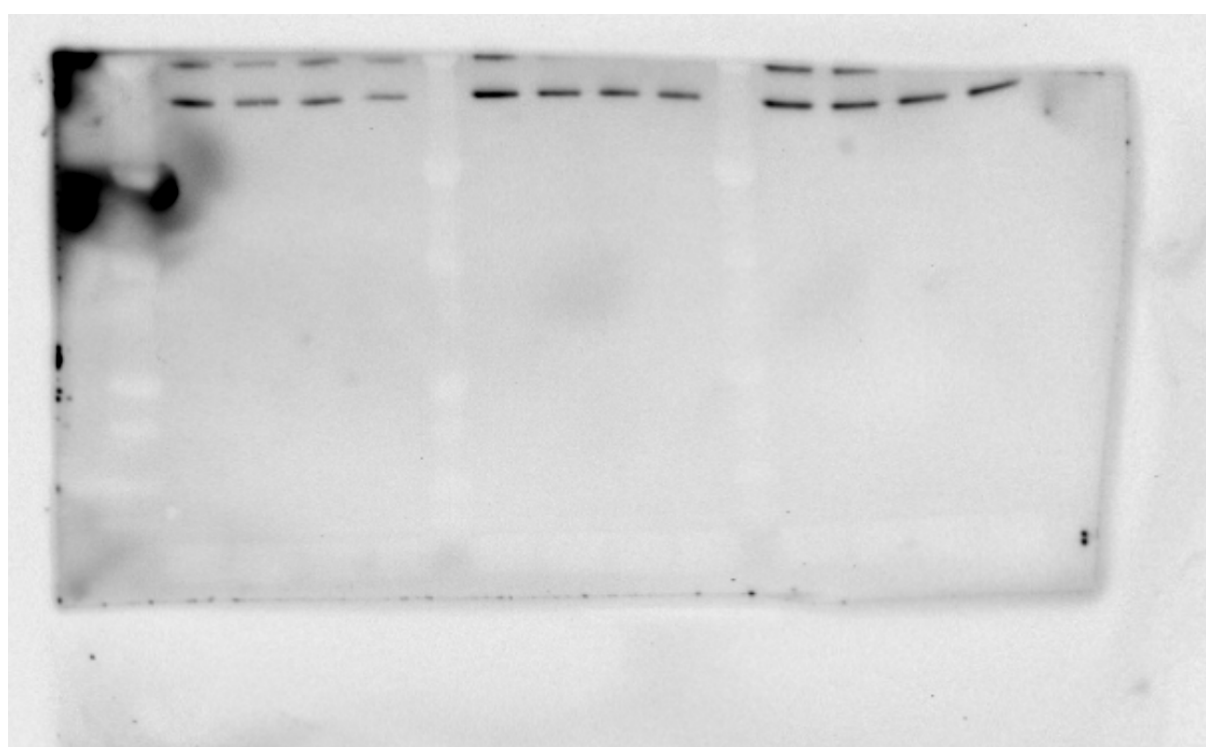

Supplement: Supplementary file 8 — Unprocessed blots. [file 41588_2024_1948_MOESM8_ESM.pdf]

Extended Data Figure 5a

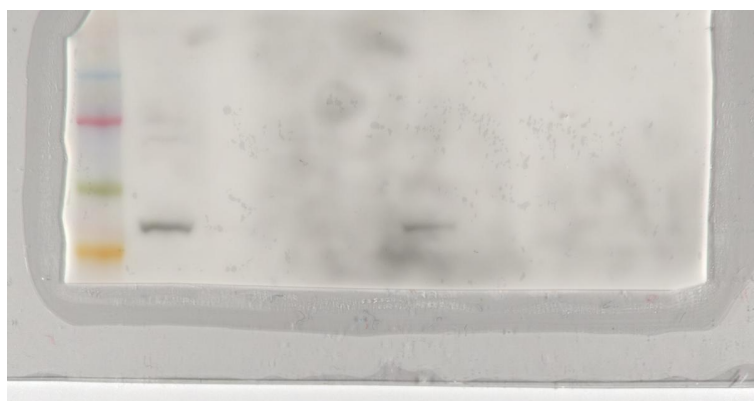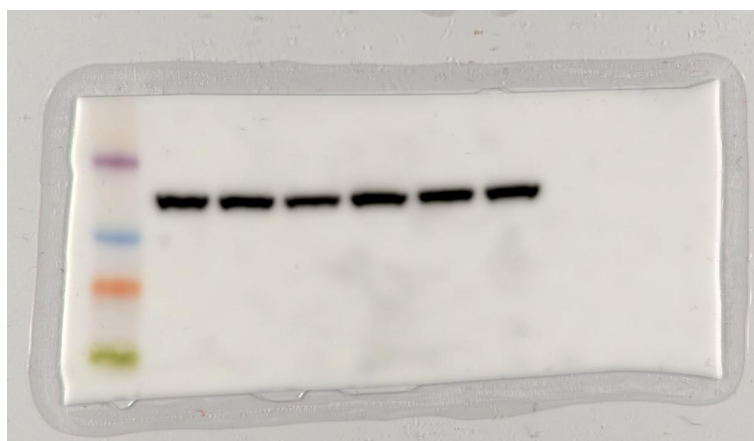

Supplement: Supplementary file 9 — Unprocessed blots. [file 41588_2024_1948_MOESM9_ESM.pdf]
